# Comparing methods to predict baseline mortality for excess mortality calculations

**DOI:** 10.1186/s12874-023-02061-w

**Published:** 2023-10-18

**Authors:** Tamás Ferenci

**Affiliations:** 1 Physiological Controls Research Center, Obuda University, Budapest, Hungary; 2 Department of Statistics, Corvinus University of Budapest, Budapest, Hungary; 3 National Laboratory for Health Security, Budapest, Hungary

**Keywords:** Excess mortality, Spline regression, Prediction, Robustness

## Abstract

**Background:**

The World Health Organization (WHO)’s excess mortality estimates presented in May 2022 stirred controversy, due in part to the high estimate provided for Germany, which was later attributed to the spline model used. This paper aims to reproduce the problem using synthetic datasets, thus allowing the investigation of its sensitivity to parameters, both of the mortality curve and of the used method, thereby shedding light on the conditions that gave rise to this error and identifying possible remedies.

**Methods:**

A negative binomial model was used accounting for long-term change, seasonality, flu seasons, and heat waves. Simulated mortality curves from this model were then analysed using simple methods (mean, linear trend), the WHO method, and the method of Acosta and Irizarry.

**Results:**

The performance of the WHO’s method with its original parametrization was indeed very poor, however it can be profoundly improved by a better choice of parameters. The Acosta–Irizarry method outperformed the WHO method despite being also based on splines, but it was also dependent on its parameters. Linear extrapolation could produce very good results, but was highly dependent on the choice of the starting year, while the average was the worst in almost all cases.

**Conclusions:**

Splines are not inherently unsuitable for predicting baseline mortality, but caution should be taken. In particular, the results suggest that the key issue is that the splines should not be too flexible to avoid overfitting. Even after having investigated a limited number of scenarios, the results suggest that there is not a single method that outperforms the others in all situations. As the WHO method on the German data illustrates, whatever method is chosen, it remains important to visualize the data, the fit, and the predictions before trusting any result. It will be interesting to see whether further research including other scenarios will come to similar conclusions.

**Supplementary Information:**

The online version contains supplementary material available at 10.1186/s12874-023-02061-w.

## Background

Excess mortality is the difference between the actual all-cause mortality (number of deaths) over a particular time period in a given country or (sub- or supranational) region and its “expected” mortality, which refers to the mortality statistically forecasted from the region’s historical data. Excess mortality calculations can be used to characterize the impact of an event on mortality if the data were obtained before the onset of the event. Therefore, the prediction pertains to a counterfactual mortality that would have been observed without the event [1]. Excess mortality can thus measure the impact of the event, assuming that the prediction is correct.

Calculating excess mortality is particularly useful if the event’s impact on mortality is difficult to measure directly. For instance, one of the typical applications is characterizing the mortality associated with natural disasters [2–4], but it is also used for epidemics, such as the seasonal flu, where direct mortality registration is missing, incomplete, or unreliable [5, 6].

Coronavirus disease 2019 (COVID-19) is no exception. While the mortality attributed to COVID-19 is reported in developed countries, typically daily or weekly, two drawbacks in the reporting have been realized. First, the number of reported deaths is – while to much less extent than the number of reported cases – still contingent on testing activity, which may be vastly different between countries or time periods. Second, despite efforts at standardization, the criteria for death certification may be different between countries [7]. Excess mortality resolves both of these problems because it is completely exempt from differences in testing intensity and cause-of-death certification procedures. This makes it especially suitable for between-country comparisons, which are critical to better understand the pandemic, particularly with regard to evaluating different control measures and responses [8].

This, however, comes at a price. First, and perhaps most importantly, excess mortality is inherently a gross metric, measuring both direct and indirect effects; the latter of which can be both positive (e.g., COVID-19 control measures also protect against the flu) and negative (e.g., the treatment of other diseases becomes less efficient) [9]. Second, excess mortality is the slowest indicator. The necessary data (i.e., the number of deaths) usually becomes available after 4 weeks (and even that is typically revised to some extent later) even in developed countries. This is in contrast to reported COVID-19 deaths, which are available by the following week or even the next day. Finally, the whole calculation depends on how accurate the forecast was.

The last of these issues is the focus of the current study. Given the importance of cross-country comparisons, the results should reflect true differences and should not be too sensitive to the prediction method used.

Only those methods that use the traditional regression approach are considered here. Methods using ARIMA models [10–13], the Holt–Winters method [14], or those based on Gaussian process [15] are not considered, nor are ensemble methods [16, 17]. Questions concerning age or sex stratification or standardization [18], small area estimation [19, 20], and the inclusion of covariates (e.g., temperature) to improve modelling [16, 17, 19] are also not considered.

These considerations leave us with two matters of concern: the handling of seasonality and the handling of a long-term trend. For the latter, the following are the typical solutions concerning COVID-19:

- Using the last prepandemic year [16, 21]. This solution is good – even if not perfect –because it uses data closest to the investigated period. However, this metric has a huge variance due to the natural year-to-year variability in mortality.
- Using the average of a few prepandemic years (typically 5 years) [22–28]. This is more reliable than the previous solution because averaging reduces variability. However, it is even more biased in case the mortality has a long-term trend (which it almost always has). For instance, if mortality is falling, this provides an overestimation; thus, excess mortality is underestimated.
- Using a linear trend extrapolation [29–31]. This approach accounts for the potential trends in mortality, removing the bias of the above-mentioned methods, at least as far as linearity is acceptable, but it depends on the selection of the starting year from which the linear curve is fitted to the data.
- Using splines [32, 33]. The method of Acosta and Irizarry [34, 35] is based on splines, just as many other custom implementations [16, 36], which, crucially, includes the model used by the World Health Organization (WHO) [37].

While this issue has received minimal public attention, the choice of the method (and the parameters) used to handle long-term trends may have a highly relevant impact on the results of the calculation. This is evidenced by the case of excess mortality estimation by the WHO. On May 5, 2022, the WHO published its excess mortality estimates [38], which immediately raised several questions. In particular, it was noted that the estimates for Germany were surprisingly high [39]: the WHO estimated that 195,000 cumulative excess deaths occurred in Germany in 2020 and 2021, a figure that was inexplicably larger than every other previous estimate. For instance, the World Mortality Dataset reported 85,123 excess deaths in Germany for the same period [1].

**Fig. 1:**
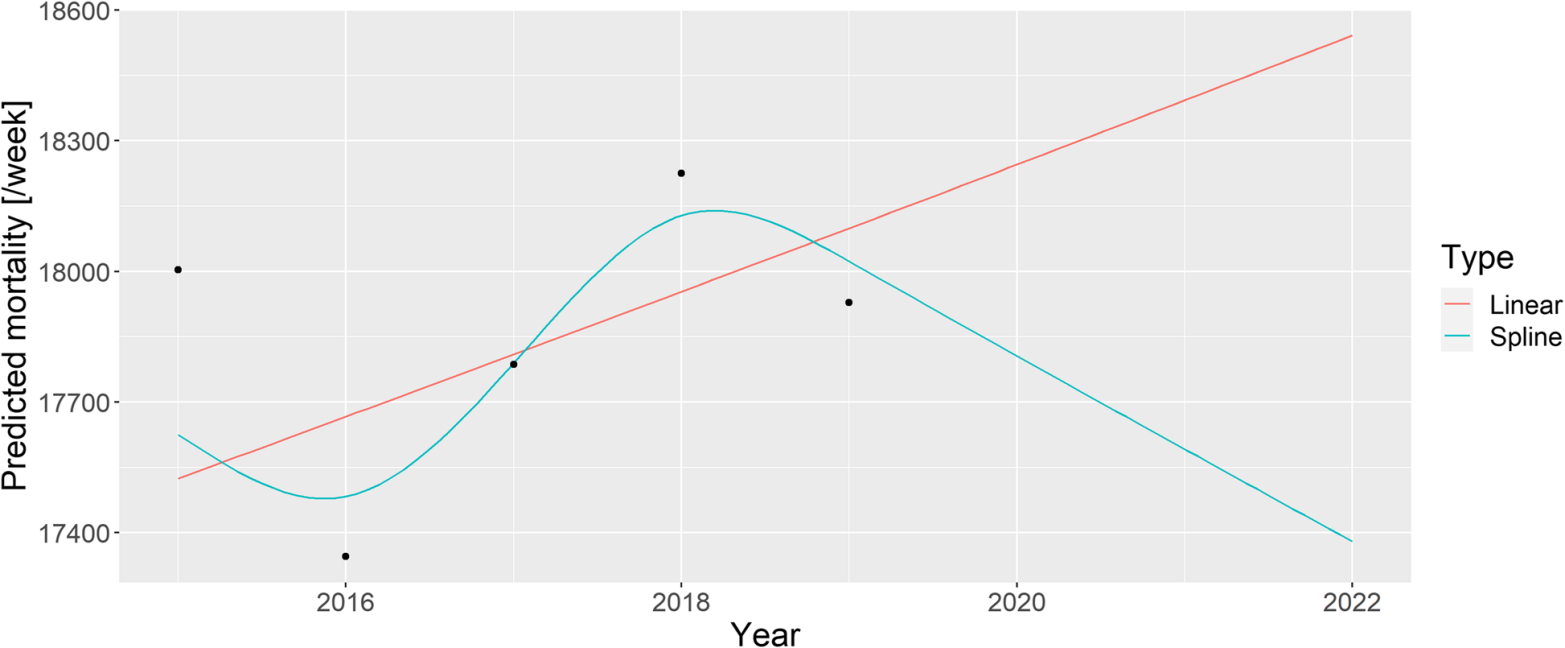
A linear trend and a spline fitted on the 2015–2019 German mortality data and extrapolated to 2020 and 2021

The case was so intriguing that one paper termed it the “German puzzle” [39]. Figure 1 illustrates the “German puzzle” using actual German data, with the curves fitted on the 2015–2019 data and extrapolated to 2020 and 2021 (as done by the WHO). While the dots visually indicate a rather clear simple upward trend (as shown by the linear extrapolation), the spline prediction turns back.

The WHO [37] later explained that the problem resulted from two issues. First, the WHO rescaled the raw data to compensate for underreporting (caused by late registration, for instance), but this approach was unnecessary in the case of Germany, which had excellent death registration. Figure 1 shows the unadjusted German data avoiding this problem to focus on the second issue; that is, the usage of splines, which is the subject of the current investigation.

As described above, the WHO method uses a spline to capture the long-term trend. However, the lower data of 2019 had a very high impact on the spline, and this single observation turned the entire spline despite earlier points showing an upward trend. Too much weight seems to have been placed on this – likely short-term, random, noise-like – fluctuation; hence, the extrapolation was too sensitive for this. The culprit was quickly identified as the spline regression itself, with one commentator saying that “[e]xtrapolating a spline is a known bad practice” [39].

However, questions have emerged as to whether splines are really to be blamed. Motivated by the intention to better understand the “German puzzle,” this study aims to investigate the following questions: 1) Are splines really the culprit *per se*?, 2) What were the particular characteristics, both of the scenario and of the used spline regression used that gave rise to the problem?, and 3) Is there a better way to predict the baseline for the excess mortality calculation to avoid this problem?

To answer these questions, we first needed to devise a model that could generate mortality curves that capture the relevant features exhibited by the real-life German example. Thus, calculating the accuracy of a forecast would be possible because the ground truth is now known, and we could investigate how the parameters of the simulation influence it. By averaging several simulations, the mean accuracy can be approximated, allowing for a comparison of the methods and investigation of its dependence on the parameters – both for the mortality curve and for the method – thereby hopefully resolving the “German puzzle.”

This investigation focuses on the errors of prediction. However, as excess mortality is defined as the difference between actual and predicted mortality, any error in the prediction directly translates to the same error in the estimation of excess mortality (given any actual mortality). Thus, the current study equivalently covers the errors in the estimation of excess mortality itself.

It should be noted that the present study does not use age or sex stratification or consider the size and composition of the background population. While these parameters are important, the WHO’s original study also did not take these factors into account.

## Methods

### Data source

Data on weekly all-cause mortalities for Germany were obtained from the European Statistical Office (Eurostat) database demo_r_mwk_ts. We applied no additional pre-processing or correction such as that for late registration, as part of the problem with the WHO’s approach was caused by upscaling, and we wanted to focus solely on the modelling aspect. Additional file 1 shows a detailed comparison of the possible data sources.

**Fig. 2:**
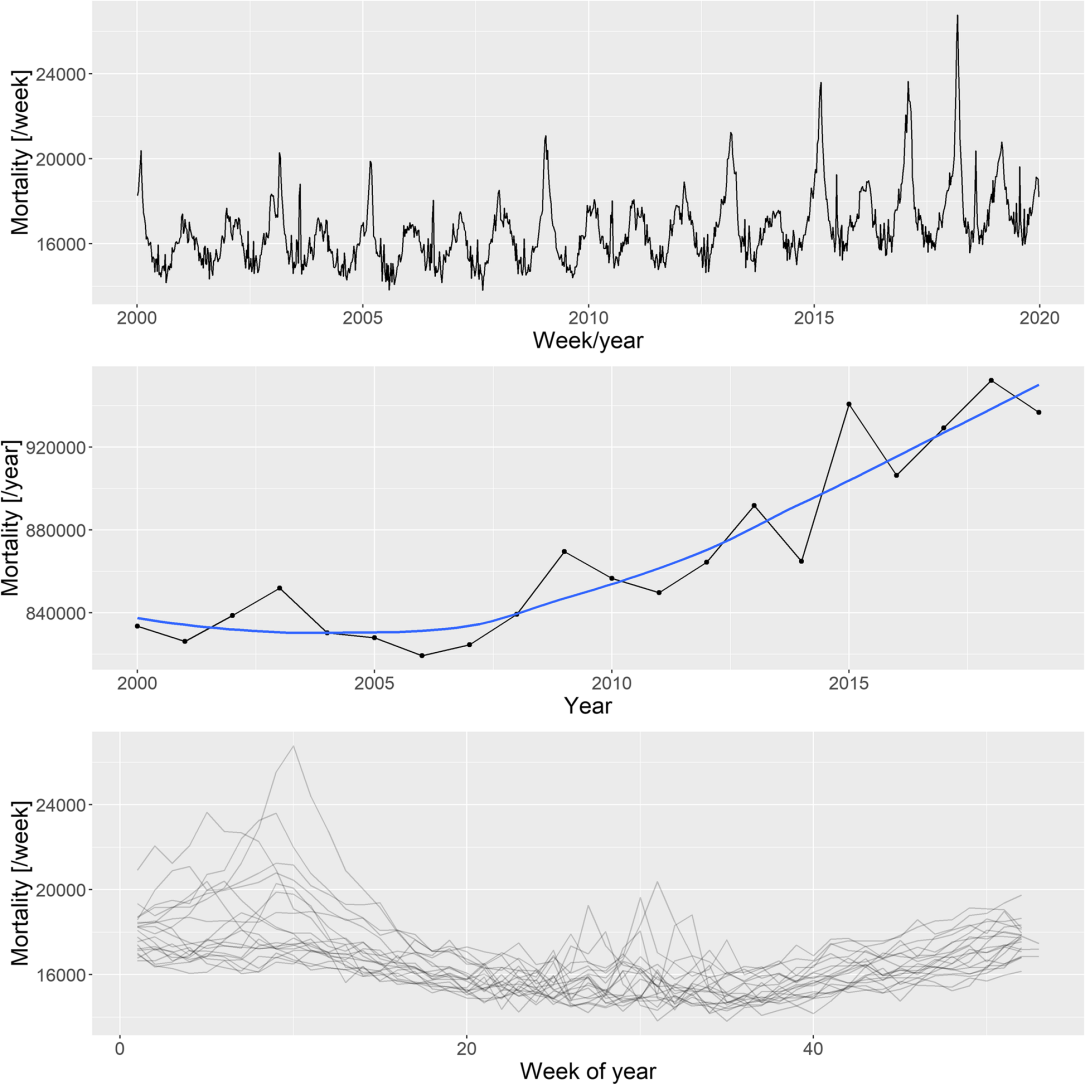
Weekly mortalities (upper), yearly mortalities with the LOESS-smoother (middle), and the seasonal pattern (bottom) for the German mortality data, 2000–2019

Figure 2 illustrates the basic properties of the data (raw weekly values, yearly trend, and seasonal pattern).

### Simulation model

Based on the patterns shown in Fig. 2, the following three components were used to create the synthetic datasets:

- Long-term change, modelled with a quadratic trend; described by three parameters (constant, linear, and quadratic terms)
- Deterministic seasonality, modelled with a single harmonic (sinusoidal) term; described by two parameters (amplitude and phase)
- Random additional peaks during winter (i.e., flu season) and summer (i.e., heat waves); described in each season by five parameters (probability of the peak, minimum and maximum values of the peak height, and minimum and maximum values of the peak width)

These components governed the expected value; the actual counts were obtained from a negative binomial distribution with a constant size and log link function.

Thus, the number of deaths at time *t* (*D*_*t*_) was simulated according to a negative binomial model *D*_*t*_ ~ *NegBin(μ*_*t*_*, s)*, with mean *μ*_*t*_ and size parameter *s* = *1000*. The mean was modelled such that log*(μ*_*t*_*)* = *β*_*0*_ + *β*_*1*_* · t* + *β*_*2*_* · t*^*2*^ + *A ·* cos*(2π · w[t]* + *φ)*, where *w[t]* is the week of time *t*, scaled from 0 to 1. The first three terms in this equation refer to the “long-term trend” (characterized by parameters *β*_*0*_ = 10.11, *β*_*1*_ =  − 7.36*·10*^*–*5^, and *β*_*2*_ = 3.04 · 10^*–*9^), and the latter to the seasonality (characterized by parameters *A* = 0.07 and *φ* =  − 0.61). Additionally, some peaks have been randomly added to log*(μ*_*t*_*)*, with widths between 8.41 and 35.36 and heights between 0.11 and 0.33 positioned in the winter (uniformly between weeks 1 and 11) with 0.45 probability in each year, and with widths between 0.86 and 9.24 and heights between 0.10 and 0.24 positioned in the summer (uniformly between weeks 26 and 37) with 0.40 probability in each year. The shape of the peaks follows the probability density function of the Cauchy distribution. These parameters were chosen so that the simulated curves mimic the properties of the real-life mortality curve.

Additional file 2 describes in detail how the above-mentioned model is built (including the estimation from the actual German data that resulted in these parameters, and an example of the simulated data along with the real data).

### Baseline mortality prediction

We predicted mortality by using four methods: the WHO method, an advanced alternative method developed by Acosta and Irizarry in 2020 that also uses splines [34], and two simple methods for comparison. These latter methods cover classical statistical methods that are widely used for predicting baseline mortality in excess mortality studies. The methods are detailed below:

- Average: after accounting for seasonality with a single cyclic cubic regression spline, the average of the preceding years was used as the constant, predicted value. The response distribution is assumed to be negative binomial (with the overdispersion parameter estimated from the data) with a log link function. Parameter: starting year (i.e., how many previous years were used for averaging). Some studies used the last prepandemic year (2019) as the predicted baseline mortality, this is just the special case of this method, with the starting year set to 2019.
- Linear: after accounting for seasonality with a single cyclic cubic regression spline, the long-term trend was modelled with a linear trend. The response distribution is assumed to be negative binomial (with the overdispersion parameter estimated from the data) with a log link function. Parameter: starting year (from which the model was fitted).
- WHO method: this method was reconstructed according to the description mentioned above [37]. Briefly, seasonality was accounted for with a single cyclic cubic regression spline (as done in previous cases), and the long-term trend was accounted for with a thin plate regression spline. The only deviation compared with WHO’s study is that the actual time (number of days since January 1, 1970) was used as the predictor of the long-term trend, not the abruptly changing year. The response distribution is assumed to be negative binomial (with the overdispersion parameter estimated from the data) with a log link function, and the model was estimated with restricted maximum likelihood. Second derivative penalty was used for constructing the spline; thus, the forecasting would be a linear extrapolation. Parameters: starting year (from which the model is fitted) and $k$, which is the dimension of the basis of the spline used for capturing the long-term trend.
- Acosta–Irizarry (AI) method: the method described in [34] using their reference implementation. It offers many advantages when estimating excess mortality, however, these advantages partly appear only in the second stage (i.e., calculating the excess after the expected is forecasted). Regarding baseline prediction, the method is similar to that of the WHO in using splines, with three differences. First, for capturing seasonality, two harmonic terms are used (with prespecified frequencies of 1/365 and 2/365 as default, and arbitrary phase estimated from the data) instead of the cyclic cubic regression spline. Second, the spline to capture the long-term trend is a natural cubic spline, not a thin plate regression spline, with the number of knots selectable. If the number of years in the training data is less than 7, a linear trend is used instead of the spline. Finally, the response distribution is quasi-Poisson (with a log link function). Parameters: starting year (from which the model is fitted) and $tkpy$, which denotes the number of trend knots per year. Other parameters are left on their default values (i.e., two harmonic terms are used).

The equations presented in Table 1 provide an overview of these modelling approaches.

**Table 1:** Overview of the models used to create predictions

**Name**	**Model**
Average	${Y}_{t} \sim NegBin\left({\mu }_{t},\theta \right)$ $\mathrm{log}\left({\mu }_{t}\right)= {\beta }_{0}+{f}_{cc}\left(w\left[t\right]\right)$
Linear	${Y}_{t} \sim NegBin\left({\mu }_{t},\theta \right)$ $\mathrm{log}\left({\mu }_{t}\right)= {\beta }_{0}+{\beta }_{1}t+{f}_{cc}\left(w\left[t\right]\right)$
WHO	${Y}_{t} \sim NegBin\left({\mu }_{t},\theta \right)$ $\mathrm{log}\left({\mu }_{t}\right)= {f}_{tp}\left(t\right)+{f}_{cc}\left(w\left[t\right]\right)$
Acosta–Irizarry	$${Y}_{t} \sim QuasiPoi\left({\mu }_{t}\right)$$ $$\mathrm{log}\left({\mu }_{t}\right)=\left\{\begin{array}{c} {\beta }_{0}+{f}_{nc}\left(t\right)+{\sum }_{k=1}^{2}\left[\mathrm{sin}\left(2\pi \cdot k\cdot w\left[t\right]\right)+\mathrm{cos}\left(2\pi \cdot k\cdot w\left[t\right]\right)\right]*\\ {\beta }_{0}+{\beta }_{1}t+{\sum }_{k=1}^{2}\left[\mathrm{sin}\left(2\pi \cdot k\cdot w\left[t\right]\right)+\mathrm{cos}\left(2\pi \cdot k\cdot w\left[t\right]\right)\right]**\end{array}\right.$$

*cc* cyclic cubic regression spline, *tp* thin plate regression spline, *nc* natural cubic spline, *: > 7 years training data, **: ≤ 7 years training data, *w[t]*: week of the year scaled to 0–1

As already noted, population size is not included in the models, consistent with what the WHO did for the analysis of countries with frequently available data. Thus, changes in the population size are absorbed into the changes in death counts without explicit modelling, clearly suggesting potential room for improvement.

### Validation through simulation

First, a synthetic dataset was randomly generated from the model described above using the investigated parameters (parameters of the scenario). This dataset simulated mortalities recorded from 2000 to 2023. We then applied the investigated prediction method with the investigated parametrization (parameters of the method), and after fitting on the 2000–2019 data, we obtained a prediction for 2020–2023, where it can be contrasted with the simulation’s actual values, which represent the ground truth in this case. Denoting the actual number of deaths in the simulated dataset for year *y* with ${M}_{y}={\sum }_{t:y\left[t\right]=y}{D}_{t}$ and the predicted number with ${\widehat{M}}_{y}$, the goodness of prediction is quantified with mean squared error (*MSE* = $\frac{1}{4}{\sum }_{y=2020}^{2023}{\left({M}_{y}-{\widehat{M}}_{y}\right)}^{2}$), mean absolute percentage error (*MAPE* = $\frac{1}{4}{\sum }_{y=2020}^{2023}\left|\frac{{M}_{y}-{\widehat{M}}_{y}}{{M}_{y}}\right|$), and bias (*Bs* = $\frac{1}{4}{\sum }_{y=2020}^{2023}\left({M}_{y}-{\widehat{M}}_{y}\right)$). This whole procedure was repeated 1000 times, and the metrics were averaged over these replications. This step was then repeated for all prediction methods, all parameters of the method, and all parameters of the scenario.

The investigated parameters of the prediction methods were the following:

- Average: starting years 2000, 2005, 2010, 2015, and 2019
- Linear: starting years 2000, 2005, 2010, and 2015
- WHO method: all possible combinations of the starting years 2000, 2005, 2010, and 2015 and $k$ (basis dimension) 5, 10, 15, and 20
- AI method: all possible combinations of the starting years 2000, 2005, 2010, and 2015 and *tkpy* (trend knots per year) 1/4, 1/5, 1/7, 1/9, and 1/12

For the scenario, simulations were run with the optimal parameters to mimic the real-life German situation, as discussed previously (base case scenario), and three further parameter sets, describing a scenario where the long-term trend is linear (*β*_*0*_ = 10.11, *β*_*1*_ =  − 7.36 · 10^*–*5^ and *β*_*2*_ = 0*)*, constant (*β*_*0*_ = 10.11, *β*_*1*_ = 0 and *β*_*2*_ = 0*)*, and when it is non-monotone (*β*_*0*_ = 10, *β*_*1*_ = 9.5 · 10^*–*5^ and *β*_*2*_ =  − 3*·10*^*–*9^*).*

This framework also enables us to investigate any further scenarios, including varying parameters other than the long-term trend, or varying several in a combinatorial fashion (although the latter has a very high computational burden).

Additional file 3 details the simulation.

### Programs used

All calculations were performed using the R statistical program package version 4.2.2 with the packages data.table (version 1.14.4), ggplot2 (version 3.4.0), excessmort (version 0.6.1), mgcv (version 1.8.41), scorepeak (version 0.1.2), parallel (version 4.2.2) lubridate (version 1.8.0), ISOweek (version 0.6.2), and eurostat (version 3.7.10).

The full source code allowing for complete reproducibility is openly available at: https://github.com/tamas-ferenci/MortalityPrediction.

## Results

**Fig. 3:**
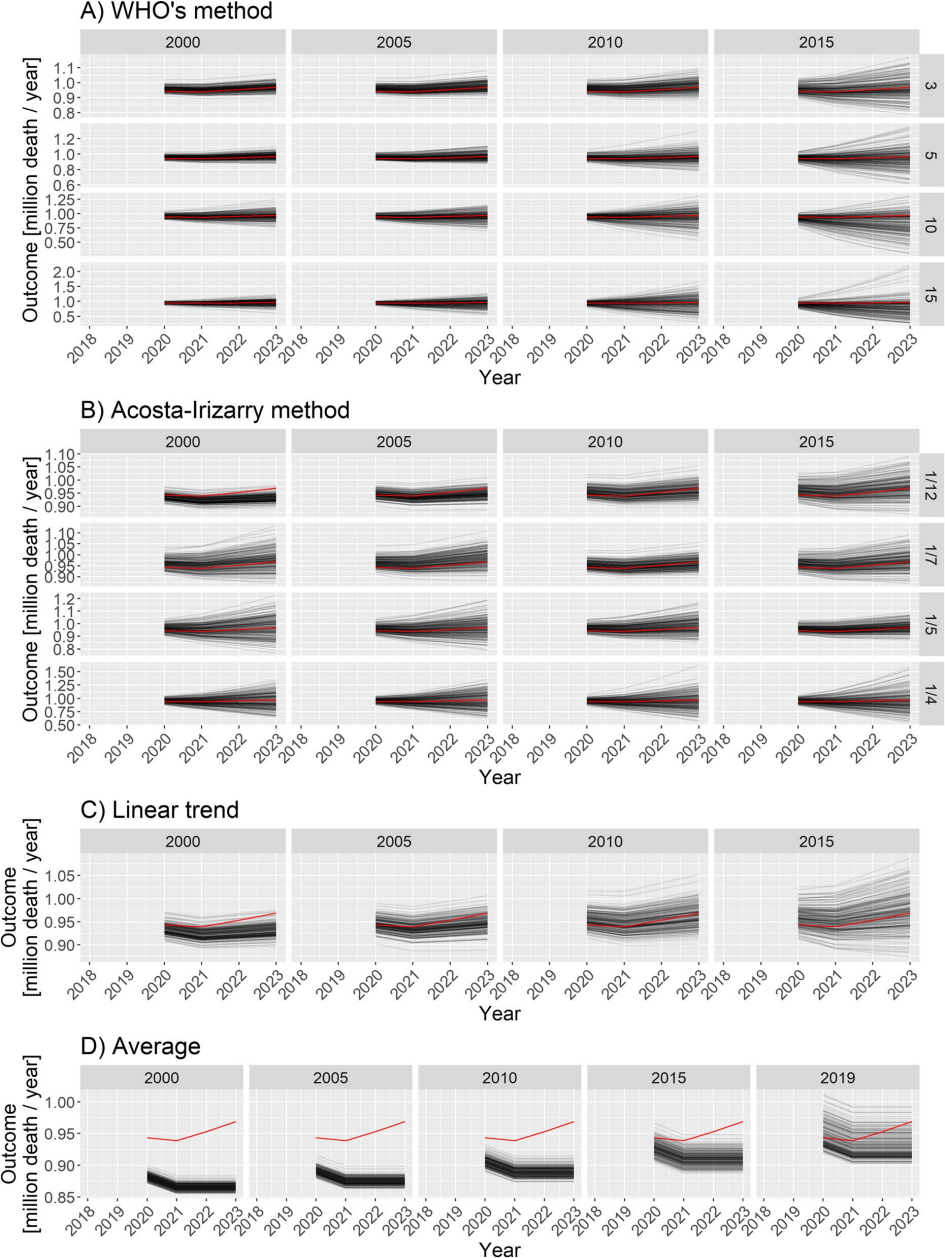
Estimated yearly deaths (for 2020–2023) for 200 randomly selected simulations (black lines) together with the ground truth (red line). **A** WHO method, **B** Acosta–Irizarry method, **C** Linear trend, **D** Average. Parameters of the methods are shown in column and row headers, and parameters of the scenario are set to the base case values. Note that 2020 is a long year with 53 weeks; therefore, higher values are expected for that year

Figure 3 illustrates the 2020–2023 predictions for the base case scenario by showing the estimated yearly deaths for 200 randomly selected simulations together with the ground truth for all four methods with all possible parameters.

**Fig. 4:**
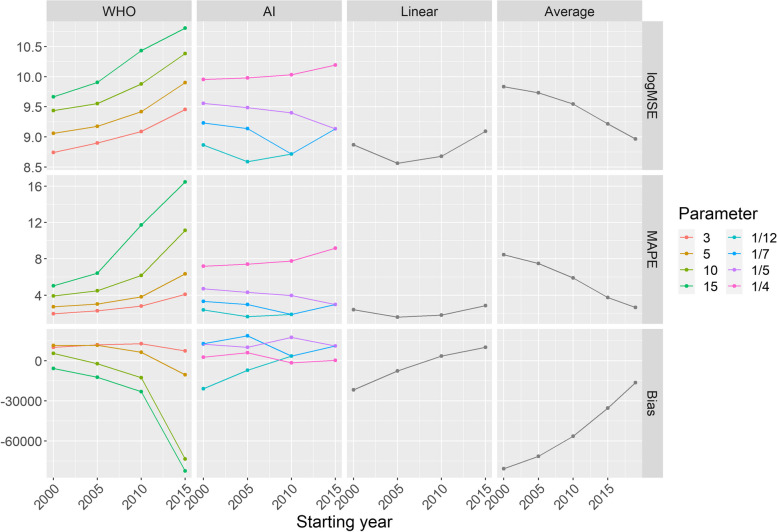
Different error metrics (logMSE, MAPE, and Bias) for all methods and all possible parameter combinations of all methods. Parameters of the scenario are set to the base case values

Figure 3 already strongly suggests some tendencies but, for a precise evaluation, we needed to calculate the error metrics. Figure 4 shows the three error metrics for all methods and all possible parametrizations. As shown in the figure, the ordering of the methods according to different criteria is largely consistent.

As suggested in Fig. 3, this confirms that $k=3$ (WHO) and *tkpy* = 1/12 or 1/7 (AI) are the best parameters in this particular scenario. Note that the default value in the method used by the WHO is *k* = 10, but it is just $tkpy=1/7$ for the AI method. Figures 3 and 4 shed light on the nature of error. The linear trend and average methods are particularly clear in this respect: early starting ensured low variance, but it was highly biased. Conversely, later starting reduced the bias but increased the variance. Thus, this observation is a typical example of a bias-variance trade-off.

**Fig. 5:**
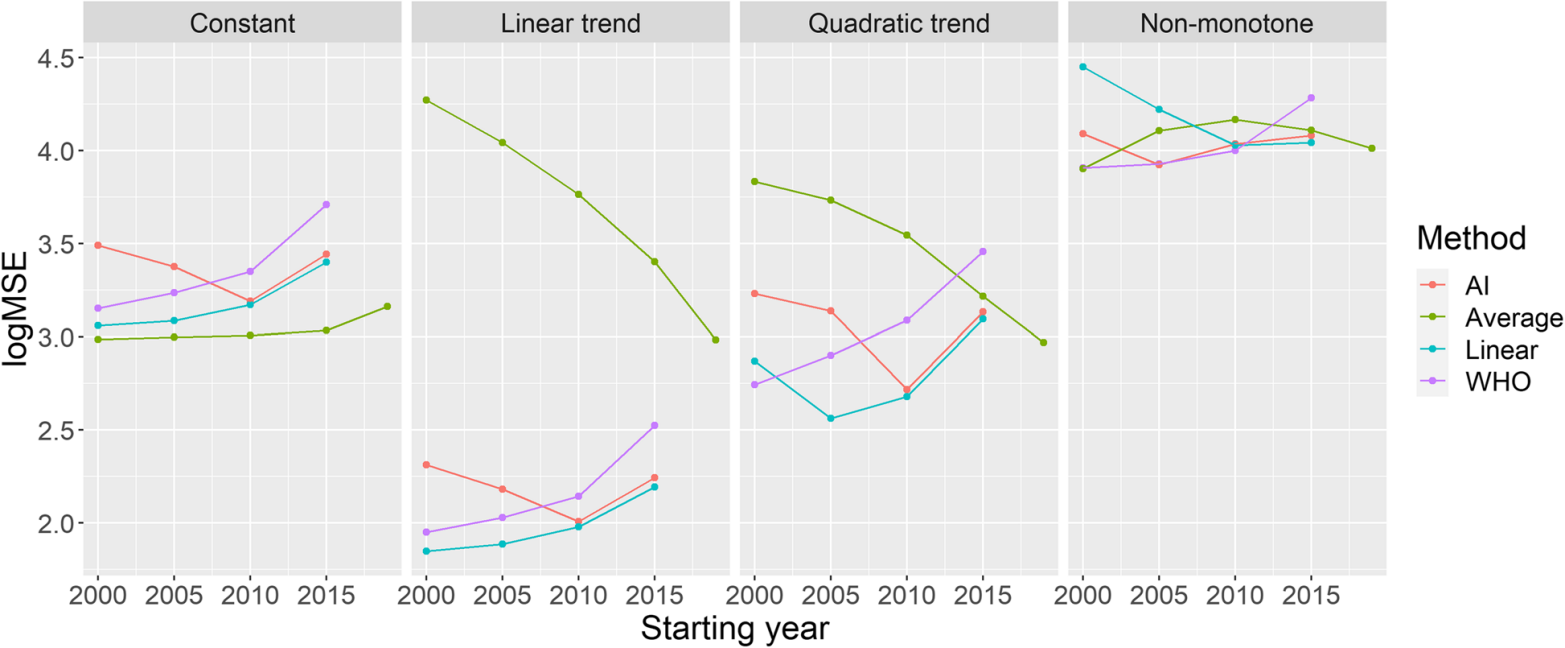
Mean squared errors of the investigated methods by starting year on a logarithmic scale (with k = 3 for the WHO method and tkpy = 1/7 for the AI approach) for the four defined scenarios

All the above-mentioned investigations used the base case scenario for the simulated mortality curve. Figure 5 shows the MSEs achievable with each method in the further investigated scenarios representing four distinct types of the long-term trend of simulated mortality as a function of the starting year (with *k* = 3 for the WHO method and *tkpy* = 1/7 for the AI approach).

Table 2 summarizes the error metrics of all four methods.

**Table 2:** Mean squared errors of the investigated methods, using the parametrization with the lowest MSE for each scenario (data shown as mean ± standard deviation)

**Method**	**Constant**	**Linear trend**	**Quadratic trend**	**Non-monotone**
AI	1182.2 ± 1273.6	73.3 ± 77.4	388.2 ± 553.5	8380.2 ± 14478
Average	969.0 ± 596.0	961.3 ± 753.1	929.6 ± 1039.6	8013.8 ± 4529.7
Linear	1150.0 ± 1213.7	70.3 ± 73.1	365.5 ± 524.0	10,702.6 ± 11901.7
WHO	1418.4 ± 2075.1	89.1 ± 123.8	552.5 ± 824.5	8067.2 ± 9866.4

Finally, considering that different methods were evaluated on the same simulated dataset for each simulation, we could directly compare not only the averages but also the errors themselves. Additional file 4 presents this possibility.

## Discussion

The results of this study demonstrate that we could reliably reproduce the “German puzzle” using synthetic datasets. This approach allowed us to investigate how the results depend on the method used, its parameters, and the parameters of the scenario.

As expected, prediction with averaging had the highest error, except for very poor parametrizations of the spline-based methods, and it was highly biased. Its performance was improved by a later starting year; that is, a smaller bias offsets the larger variability. Naturally, it performs well in a practically very unlikely case when even true mortality is constant.

Linear extrapolation seemed to be a very promising alternative, comparable to the considerably more sophisticated spline-based methods. The only problem was that it was very sensitive to the appropriate selection of the starting year; the phase where the change in historical mortality is linear should be covered. Linear extrapolation is prone to subjectivity and may not work at all if the linear phase is too short (thereby limiting the available information), but it depends on how wiggly the historical curve is. Naturally, it works best if the true mortality is linear, but it can perform very poorly with non-monotone curves when the starting year is not selected to match the last section that can be approximated with a linear curve, consistent with the previous remark.

Theoretically, splines can work well even in cases of non-monotone mortality. The spline-based method can use all historical data. It is not abruptly cut off as with linear extrapolation, but more weight is placed on the trends suggested by recent observations. At first glance, this method seems to be the ideal solution, delivering the benefits of linear extrapolation but without the need to “guess” the good starting point. However, as this study reveals, the definitions of “more weight” and “recent” are crucial, and certain parameter choices can result in very poor extrapolations, despite the tempting theoretical properties.

In selecting the optimal parameters, as confirmed by the results of the two spline-based methods (the WHO method and the AI method), the splines should be quite simple in baseline mortality prediction for excess mortality calculations. This finding is in line with the experiences both with the WHO method (where increasing the basis dimension decreased the performance) and the AI method (where increasing the trend knots per year decreased the performance).

One possible explanation is that mortality curves exhibit only slow changes, so high flexibility is not required. As with any regression model, excessively high model capacity can be downright detrimental, as it allows the model to pick up noise, which can cause overfitting.

Importantly, the AI method only uses splines to model the long-term trend if it has more than 7 years of data. Otherwise, it switches into a simple linear trend. This finding is completely in line with the above-mentioned remarks. That is, flexibility is useful but can backfire with small amounts of training data.

In Germany, the 2019 data were quite lower, likely because of simple random fluctuation, but the spline was flexible enough to be “able to take this bend.” Data are presented using the ISO 8601 year definition; thus, the year can be either 52 or 53 weeks long. From 2015 to 2019, every year was 52 weeks long, except for 2015, which was 1 week longer. This definition adds to the reasons why the value for 2015 is higher, increasing the German data’s wiggliness and thereby potentially contributing to the problem. The increased wiggliness of the data forces the thin plate regression spline used in the WHO method to be more flexible. (This was not a problem with the WHO’s original analysis because it used monthly data, but it could be if the WHO method is directly applied to yearly data.)

The WHO method is only acceptable if *k* ≤ 5 (but even that requires a longer observation than starting from 2015, as was done by the WHO), not higher. For the AI method, 1/4 trend knots per year was too flexible, and perhaps even 1/5 was too high. The default value in the reference implementation of the AI method is 1/7, and using a considerably higher value is not recommended. However, the WHO study did not specify what basis dimension was used [37]; nonetheless, the default of the package used is *k* = 10. Thus, even *k* = 5, and especially *k* = 3, is substantially lower. Possibly, this component is crucial in the WHO’s experience, where the starting year was 2015 (and *k* = 10 was probably used).

Between the two spline-based methods, when using rigidity parameters that are optimal in this particular scenario, the WHO method performed better with longer fitting periods, whereas the AI method performed better with shorter ones. However, the performance of the AI method was considerably less dependent on the starting year.

**Fig. 6:**
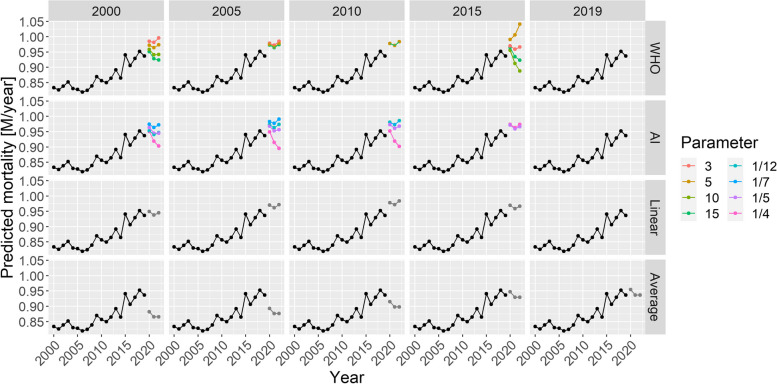
Predictions for 2020–2022 from all methods with all possible parameters, using historical German data

Perhaps one of the most important lessons learned, especially from Figs. 4 and 5, is that there is no “one size fits all” optimal choice. In other words, a method that performs well for a given true mortality curve can exhibit extremely poor performance for another shape of mortality. Moreover, the optimal choice of parameters even for one given method can substantially depend on the scenario, and a parameter that works well for one situation might be poor for another. Hence, while there are “safer choices,” recommending a universally “optimal” parameter is not possible. In selecting a good parameter for a particular case, perhaps the most important is to examine the fit of the model on historical data. Figure 6 provides an example using the German data. The figure shows the prediction for 3 years (2020–2022) from the historical data, using all methods and all parameters. A quick visual inspection immediately reveals relevant and likely meaningless predictions. The latter, unfortunately, includes that of the WHO (2015 as the starting year, *k* = *10*). Thus, such an inspection would have likely revealed the problem. Time-series bootstrap and time-series cross-validation, which are objective, might be promising alternatives for the potentially subjective method of visual inspection, still with the use of only historical data.

Two studies conducted by Nepomuceno et al. [40] and Shöley [41] are similar to ours. They used several models, partly overlapping those that are presented in our study but, importantly, neither of them considered splines for a long-term trend. Nepomuceno et al. did not try to evaluate the methods and compared these methods without having ground truth to determine any concordance. In contrast, Shöley conducted an objective evaluation but, contrary to the synthetic dataset simulation approach used in the present study, time-series cross-validation with historical data was applied to measure accuracy. Cross-validation is guaranteed to be realistic as opposed to a simulation, but it has less freedom; in this method, investigators are bound to the empirical data with limited possibilities in varying the parameters.

We did not investigate the impact of using the population and modelling death rates instead of death counts directly. We also did not examine the potential impact of data frequency. In our study, we used weekly data, which may be unavailable in developing countries but are almost universally available in developed countries. Therefore, using the most frequent data seems logical (with the appropriate handling of seasonality). Furthermore, because our focus was on developed countries, we did not consider adjusting for late registration and imputing missing data, which may be needed when full data are unavailable.

Another limitation of our study is that it only analysed point estimates. The applied prediction models can provide prediction intervals; thus, investigating their validity (e.g., coverage properties) could be a relevant future research direction.

Finally, this study did not consider the age and sex structures of the population (consistent with the WHO’s approach). However, the inclusion of these structures together with an explicit modelling of the population size might improve predictions by capturing and separating mechanisms that govern the change of population size and structure in the models. To explore whether and to which extent predictions could be improved by taking these structures into account is an important matter that requires further research.

## Conclusions

The performance of the WHO’s method with its original parametrization is indeed very poor, as revealed by extensive simulations. Thus, the “German puzzle” was not just an unfortunate mishap, but it could have been profoundly improved by selecting better parameters. The performance of the WHO method is similar to that of the AI method, with the former being slightly better for longer fitting periods and the latter for shorter ones. Despite its simplicity, linear extrapolation can exhibit a favourable performance, but this depends highly on the choice of the starting year. Conversely, the AI method exhibits relatively stable performance (considerably more stable than the WHO method) irrespective of the starting year. The performance of the average method is almost always the worst, except for very special circumstances.

This study also shows that splines are not inherently unsuitable for predicting baseline mortality, but caution should be taken, with the key issue being that the splines should not be too flexible to avoid overfitting. In general, no single method outperformed the others in the investigated scenarios. Regardless of the approach or parametrization used, it is essential to have a proper look at the data, and to visualize the fit and the predictions produced by the method used. Further research is warranted to see if these statements can be confirmed on the basis of other scenarios.

### Supplementary Information

**Additional file 1.** Comparison of data sources.

**Additional file 2.** Generating realistic synthetic datasets.

**Additional file 3.** Validation through simulation.

**Additional file 4.** Directly comparing the errors in simulation runs.

## Data Availability

The datasets generated and/or analyzed during the current study are available in the Github repository, https://github.com/tamas-ferenci/MortalityPrediction.

## Abbreviations

COVID-19: Coronavirus disease 2019
MSE: Mean squared error
MAPE: Mean absolute percentage error
WHO: World Health Organization
