# Supplementary material for: Comparing methods to predict baseline mortality for excess mortality calculations
Source: BMC Med Res Methodol. 2023 Oct 18;23:239. doi: 10.1186/s12874-023-02061-w (PMC10585880; doi:10.1186/s12874-023-02061-w)
Supplement: Supplementary file 1 — Additional file 1. Comparison of data sources. [file 12874_2023_2061_MOESM1_ESM.docx]

**Additional File 1: Comparison of data sources**

For a country like Germany, four data sources come into consideration for weekly mortality data: Eurostat [1], the Short-Term Mortality Fluctuations (STMF) dataset of the Human Mortality Database (WMD) [2], the World Mortality Database [3] and the national data provider (in this case, the Federal Statistical Office of Germany). The last is usually more complicated, limits extension to other countries and is unnecessary for developed countries, so it’ll be avoided in this case. Also, for Germany, WMD simply copies the data of the STMF (“We collect the weekly STMF data for the following countries: […] Germany, […].”) leaving us with two options.

We shall compare whether these two report identical data (Figure S1).

RawDataEurostat <- as.data.table(eurostat::get_eurostat("demo_r_mwk_ts",
 time_format = "raw"))
RawDataEurostat <- RawDataEurostat[geo=="DE"&sex=="T"]
RawDataEurostat$Year <- as.numeric(substring(RawDataEurostat$time, 1, 4))
RawDataEurostat$Week <- as.numeric(substring(RawDataEurostat$time, 6, 7))

RawDataSTMF <- fread(paste0("https://www.mortality.org/File/GetDocument/",
 "Public/STMF/Outputs/stmf.csv"))
RawDataSTMF <- RawDataSTMF[CountryCode=="DEUTNP"&Sex=="b"]

RawDataEurostatSTMF <- merge(RawDataEurostat[, .(Year, Week, ES = values)],
 RawDataSTMF[, .(Year, Week, STMF = DTotal)])

ggplot(RawDataEurostatSTMF, aes(x = ES, y = STMF)) + geom_point() +
 geom_abline(color = "red") + labs(x = "Eurostat value [/week]",
 y = "STMF value [/week]")


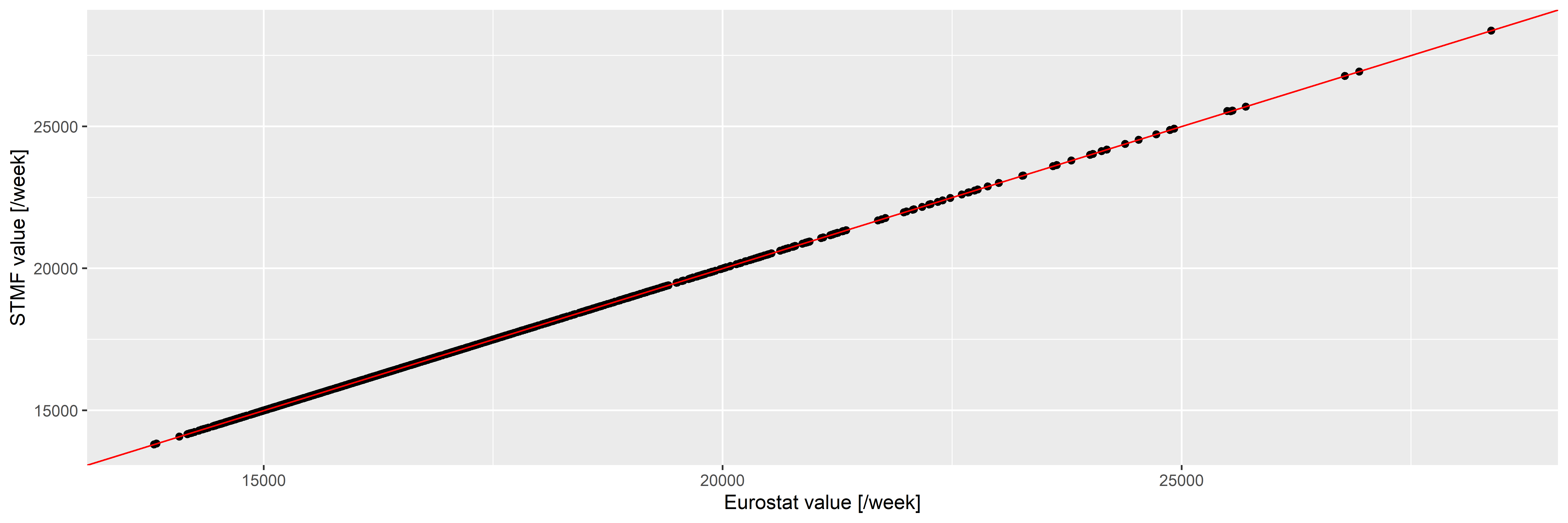


Figure S1: Weekly number of deaths according to the Eurostat (horizontal axis) and the STMF database (vertical axis) in Germany. Red line indicates the line of equality.

The two are almost identical (with a correlation of 0.9999998), with differences only occuring for the latest data and of minimal magnitude, so we can safely use the Eurostat database.

## References

1. Eurostat - Data Explorer [Internet]. [cited 2022 Jun 28]. Available from: <https://appsso.eurostat.ec.europa.eu/nui/show.do?dataset=demo_r_mwk_ts&lang=en>

2. Jdanov DA, Galarza AA, Shkolnikov VM, Jasilionis D, Németh L, Leon DA, et al. The short-term mortality fluctuation data series, monitoring mortality shocks across time and space. Sci Data [Internet]. 2021 [cited 2022 Jun 28];8:235. Available from: <https://www.nature.com/articles/s41597-021-01019-1>

3. Karlinsky A, Kobak D. Tracking excess mortality across countries during the COVID-19 pandemic with the World Mortality Dataset. eLife [Internet]. 2021 [cited 2022 Jun 28];10:e69336. Available from: <https://elifesciences.org/articles/69336>
