# Supplementary material for: Comparing methods to predict baseline mortality for excess mortality calculations
Source: BMC Med Res Methodol. 2023 Oct 18;23:239. doi: 10.1186/s12874-023-02061-w (PMC10585880; doi:10.1186/s12874-023-02061-w)
Supplement: Supplementary file 2 — Additional file 2. Generating realistic synthetic datasets. [file 12874_2023_2061_MOESM2_ESM.docx]

**Additional File 2: Generating realistic synthetic datasets**

Inspecting raw weekly values, yearly trend and seasonal pattern already gives important clues on the setup of a realistic model from which synthetic datasets could be generated. Figure S2 gives further insight by plotting each year separately.

RawData2019 <- RawData[Year<=2019]

ggplot(RawData2019, aes(x = Week, y = outcome)) + geom_line() + facet_wrap(~Year) +
 labs(x = "Week of year", y = "Mortality [/week]")


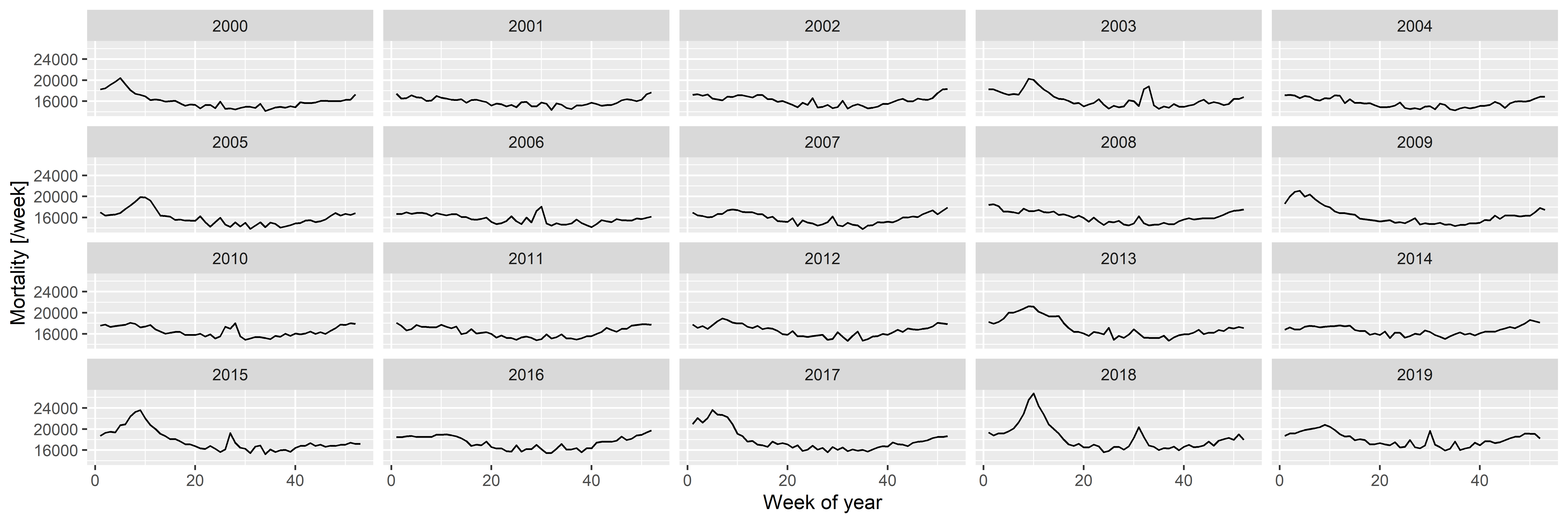


Figure S2: Weekly number of deaths in Germany, separated according to year.

The following observations can be made:

- There is a long-term trend, seemingly quadratic.
- There is a strong seasonality with winter peak and summer trough.
- There are peaks – in addition to the seasonality – in the winter and also during the summer (although the shape seems to be different, with winter peaks seeming to be broader and higher).

To investigate these, first a spline-smoothing – with thin plate regression spline [1] – is applied to obtain the long-term trend, and a single harmonic term is included as a covariate to remove seasonality. No interaction is assumed between the two, i.e., it is assumed that the seasonal pattern is the same every year. (The peaks are not accounted for at this stage which means that the curve is above the true one, but the difference is likely has minimal due to the rarity and short duration of the peaks. This will be later corrected, after the peaks were identified.) All analysis will be carried out on the log scale (meaning the effect of covariates is multiplicative) using negative binomial response distribution to allow for potential overdispersion [2].

Figure S3 shows the results, overplotted with the model where the long-term trend is a completely parametric quadratic trend. One can observe very good fit between the two, so all subsequent investigation will use the quadratic trend which is much easier to handle. This is only meaningful for short-term extrapolation, but this is what will be needed now (two years of extrapolation will be used in the present study); also it is not possible to better differentiate between functional forms at this sample size.

fitSpline <- mgcv::gam(outcome ~ s(NumTrend) + cos(2*pi*WeekScaled) +
 sin(2*pi*WeekScaled),
 data = RawData2019, family = mgcv::nb(), method = "REML")
fit <- mgcv::gam(outcome ~ poly(NumTrend, 2, raw = TRUE) + cos(2*pi*WeekScaled) +
 sin(2*pi*WeekScaled),
 data = RawData2019, family = mgcv::nb(), method = "REML")

fitTrendMinPoint <- -coef(fit)["poly(NumTrend, 2, raw = TRUE)1"]/
 (2*coef(fit)["poly(NumTrend, 2, raw = TRUE)2"])
fitTrendMinValue <- coef(fit)["(Intercept)"]-
 coef(fit)["poly(NumTrend, 2, raw = TRUE)1"]^2/(
 4*coef(fit)["poly(NumTrend, 2, raw = TRUE)2"])
fitTrend2020End <- coef(fit)["poly(NumTrend, 2, raw = TRUE)2"]*18624^2 +
 coef(fit)["poly(NumTrend, 2, raw = TRUE)1"]*18624 + coef(fit)["(Intercept)"]
fitSeasonAmplitude <- sqrt(coef(fit)["sin(2 * pi * WeekScaled)"]^2 +
 coef(fit)["cos(2 * pi * WeekScaled)"]^2)
fitSeasonPhase <- atan(-coef(fit)["sin(2 * pi * WeekScaled)"]/coef(fit)[
 "cos(2 * pi * WeekScaled)"])

predgrid <- data.frame(NumTrend = seq(min(RawData2019$NumTrend),
 max(RawData2019$NumTrend), length.out = 100),
 WeekScaled = rep(0.5, 100))
predgrid <- rbind(cbind(predgrid, Type = "Spline",
 with(predict(fitSpline, newdata = predgrid,
 newdata.guaranteed = TRUE,
 se.fit = TRUE), data.frame(fit, se.fit))),
 cbind(predgrid, Type = "Quadratic",
 with(predict(fit, newdata = predgrid, newdata.guaranteed = TRUE,
 se.fit = TRUE), data.frame(fit, se.fit))))

ggplot(predgrid, aes(x = lubridate::as_date(NumTrend), y = exp(fit),
 ymin = exp(fit - 1.96*se.fit), ymax = exp(fit + 1.96*se.fit),
 color = Type, fill = Type)) + geom_line() +
 geom_ribbon(alpha = 0.2, linetype = 0) +
 labs(x = "Date", y = "Predicted number of weekly deaths (adjusted to June-30)")


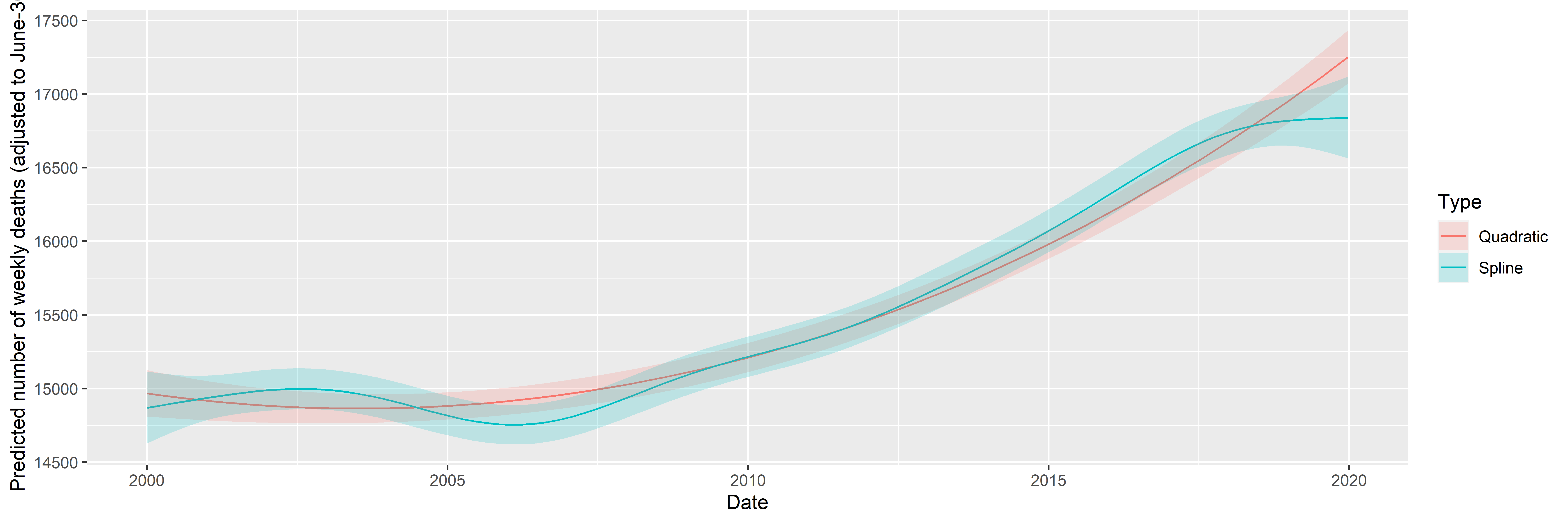


Figure S3: Fitting long-term trend as spline (black) and as quadratic trend (red); shaded area indicated 95% confidence interval. Seasonality is removed by including a single harmonic term in the regression in both cases.

The coefficients can be transformed to equivalent forms that are more meaningful. Thus, the three parameters of the quadratic trend can be expressed as a minimum point (2003-07-15), value at the minimum (15918.54) and value at the end of 2020 (18825.83). (This differs from the value seen on Figure S3, as that also includes the effect of the harmonic term.) The two parameters of the harmonic regression can be expressed as an amplitude, a multiplier (9.4%) and a phase shift (-0.7, i.e., minimum at week 32 of the year).

Figure S4 shows the predictions of the above model.

RawData2019$pred <- predict(fit)

ggplot(RawData2019, aes(x = Week, y = outcome)) +
 geom_line() + geom_line(aes(y = exp(pred)), color = "red") + facet_wrap(~Year) +
 labs(x = "Week of year", y = "Mortality [/week]")


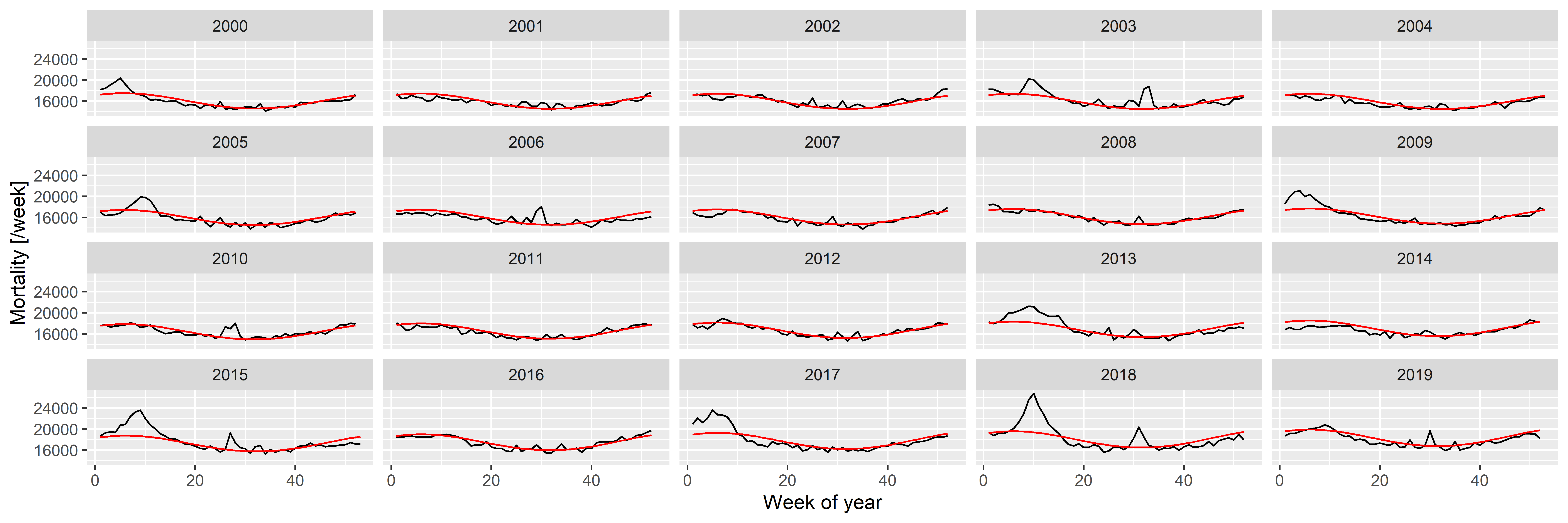


Figure S4: Weekly number of deaths in Germany, separated according to year, showing the predictions from the model with quadratic long-term trend and a single, fixed harmonic term.

A good fit can be observed, apart from summer and winter peaks. Thus, to capture them, the predictions are subtracted; with the results shown on Figure S5.

RawData2019$resid <- residuals(fit, type = "working")

peakdet <- scorepeak::detect_localmaxima(RawData2019$resid, 35) &
 scorepeak::score_type1(RawData2019$resid, 35)>0.1315
peaks <- data.table(x = RawData2019$NumTrend[peakdet], y = RawData2019$resid[peakdet])
peaks$peakDate <- lubridate::as_date(peaks$x)
peaks$Year <- lubridate::isoyear(peaks$peakDate)
peaks$peakWeek <- lubridate::isoweek(peaks$peakDate)
peaks$peakID <- 1:nrow(peaks)

ggplot(RawData2019, aes(x = Week, y = resid)) + geom_line() + facet_wrap(~Year) +
 geom_point(data = peaks, aes(x = peakWeek, y = y)) +
 labs(x = "Week of the year", y = "Working residual (log scale)")


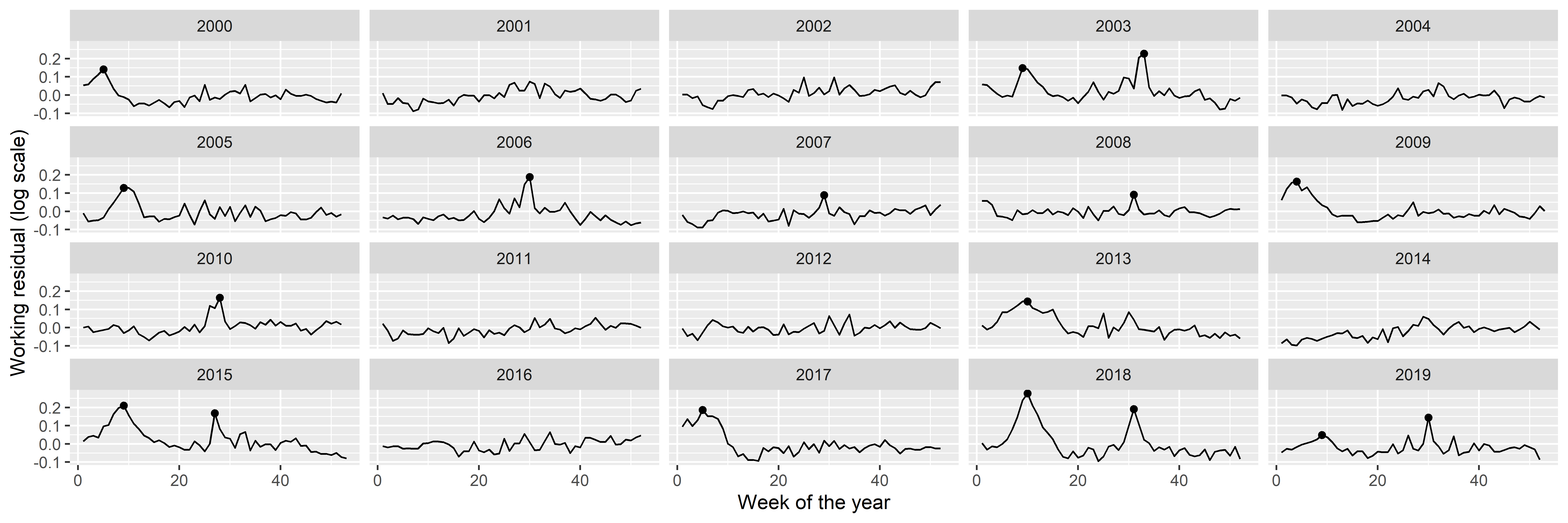


Figure S5: Residuals of the fitted model with quadratic long-term trend and a single, fixed harmonic term. Dots indicate identified peaks.

Peaks in the residuals were identified with the peak detector of Palshikar [3] using parameters that were empirically tuned to identify to visually clear peaks. Results are shown on Figure S5 with black dots; an indeed good identification of the unequivocal peaks can be seen.

Figure S6 shows the peaks themselves with a $\pm$ 100 days neighbourhood. This reinforces the idea that summer and winter peaks are somewhat different, but more importantly, suggests that the rescaled probability density function of the Cauchy distribution, i.e., $\frac{a}{\pi s}\frac{1}{1+\left( \frac{x-x_{0}}{s} \right)^{2}}+b$ might be a good – and parsimonious – function form to capture the shape of the peaks.

RawData2019$peakID <- sapply(1:nrow(RawData2019),
 function(i) which.min(abs(RawData2019$NumTrend[i]-peaks$x)))
RawData2019$peakdist <- sapply(1:nrow(RawData2019),
 function(i) RawData2019$NumTrend[i]-peaks[
 RawData2019$peakID[i],]$x)

RawData2019 <- merge(RawData2019, peaks[, .(peakID, peakWeek)])
RawData2019$peakText <- paste0(RawData2019$peakID, " (Week: ", RawData2019$peakWeek, ")")
RawData2019$peakText <- factor(RawData2019$peakText,
 levels = unique(RawData2019$peakText))

minfun <- function(x, data) sum((data$resid-(x["a"]*dcauchy(data$peakdist, x["x0"],
 exp(x["s"]))+x["b"]))^2)

RawData2019[, c("fitpeak", "x0", "s", "a", "b") :=
 with(optim(c(a = 10, x0 = 0, s = 0, b = 0), minfun,
 data = .SD[abs(peakdist)<100, .(resid = resid, peakdist)]),
 list(dcauchy(peakdist, par["x0"], exp(par["s"]))*par["a"],
 par["x0"], exp(par["s"]), par["a"],par["b"])), .(peakID)]

ggplot(RawData2019[abs(peakdist)<100], aes(x = peakdist, y = resid)) + geom_line() +
 geom_point() + facet_wrap(~peakText) + geom_line(aes(y = fitpeak + b), color = "red") +
 labs(x = "Distance from the peak [day]", y = "Working residual (log scale)")


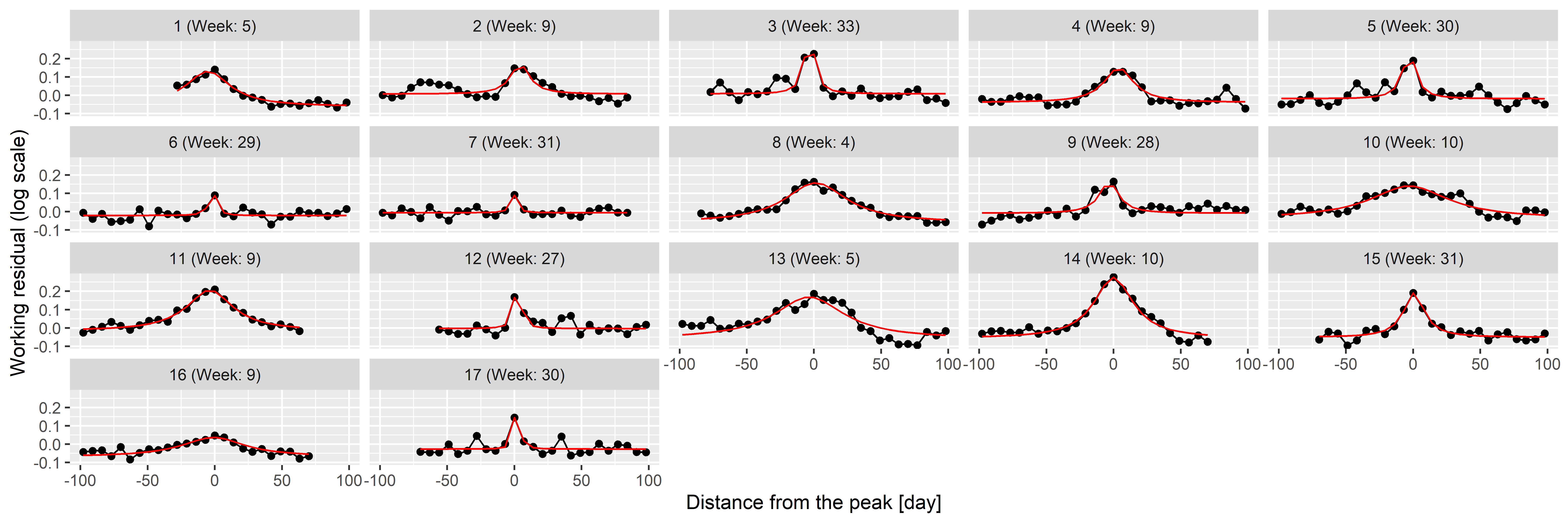


Figure S6: 100-day width neighbourhood of the identified peaks. Red line indicates the best fitting rescaled Cauchy density.

To check this theory, the best fitting function was found for each peak individually using the Nelder-Mead method [4] with mean squared error objective function. Results are shown on Figure S6 as red lines; an almost perfect fit can be observed for all peaks confirming the initial idea of using Cauchy density.

This now puts us in a position to investigate the distribution of the parameters (i.e., $a$, $b$, $s$ and $x_{0}$), which is shown on Figure S7 separated according to whether the peak is during the summer or not. Peak height is also calculated, defined as height at zero (which is $\frac{a}{\pi s\left( 1+\frac{x_{0}^{2}}{s^{2}} \right)}$) not the actual maximum height (which is at $x_{0}$) to avoid extremely large heights – which were never actually observed – due to peaks with small $s$, i.e., very narrow peaks.

peaks <- merge(peaks, unique(RawData2019[, .(peakID, x0, s, a, b)]))
peaks$Summer <- peaks$peakWeek>10 & peaks$peakWeek<35
peaks$amplitude <- peaks$a/(pi*peaks$s*(1+peaks$x0^2/peaks$s^2))

fitWinterAmplitudeMin <- min(peaks[Summer==FALSE]$amplitude)
fitWinterAmplitudeMax <- max(peaks[Summer==FALSE]$amplitude)
fitWinterSigmaMin <- min(peaks[Summer==FALSE]$s)
fitWinterSigmaMax <- max(peaks[Summer==FALSE]$s)
fitWinterProb <- sum(!peaks$Summer)/(diff(range(RawData2019$Year)) + 1)

fitSummerAmplitudeMin <- min(peaks[Summer==TRUE]$amplitude)
fitSummerAmplitudeMax <- max(peaks[Summer==TRUE]$amplitude)
fitSummerSigmaMin <- min(peaks[Summer==TRUE]$s)
fitSummerSigmaMax <- max(peaks[Summer==TRUE]$s)
fitSummerProb <- sum(peaks$Summer)/(diff(range(RawData2019$Year)) + 1)

ggplot(melt(peaks[, .(peakID, Summer, s, a, peakWeek, amplitude)],
 id.vars = c("peakID", "Summer")),
 aes(x = value, y = Summer)) + facet_wrap(~variable, scales = "free") + geom_point()


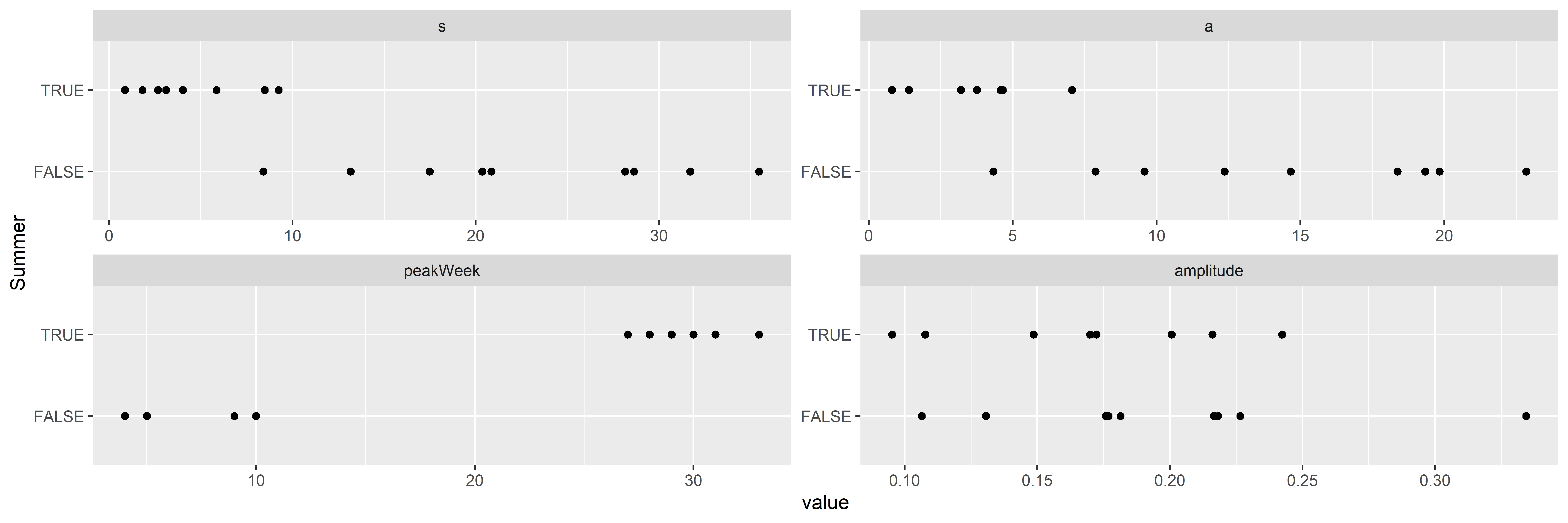


Figure S7: Distribution of the parameters of the best fitting rescaled Cauchy densities for each peak, separated according to whether the peak is during the summer.

This verifies that the width is indeed different, with the $s$ of the summer peaks being below 10, and the winter peaks being above, i.e., summer peaks are shorter in duration, raise and fall faster. Interestingly, the peak heights are not substantially different between winter and summer. Also note that the probability of having a peak at all is different: there are 8 summer peaks and 9 winter peaks (from 20 years). Winter peaks occur between weeks 4 and 10, summer peaks occur from weeks 25 to 35.

This allows the removal of the peaks (Figure S8), and, after these peaks are removed, it is possible to re-estimate trend and seasonality, now without the biasing effect of the peaks. This “bootstrap” procedure is adequate after this second iteration, as no further peaks can be seen after the removal of the re-estimated trend and seasonality.

ggplot(RawData2019, aes(x = Week, y = log(outcome))) + geom_line() + facet_wrap(~Year) +
 geom_line(aes(y = log(outcome) - fitpeak), color = "red") +
 labs(x = "Week of the year", y = "Outcome (log scale)")


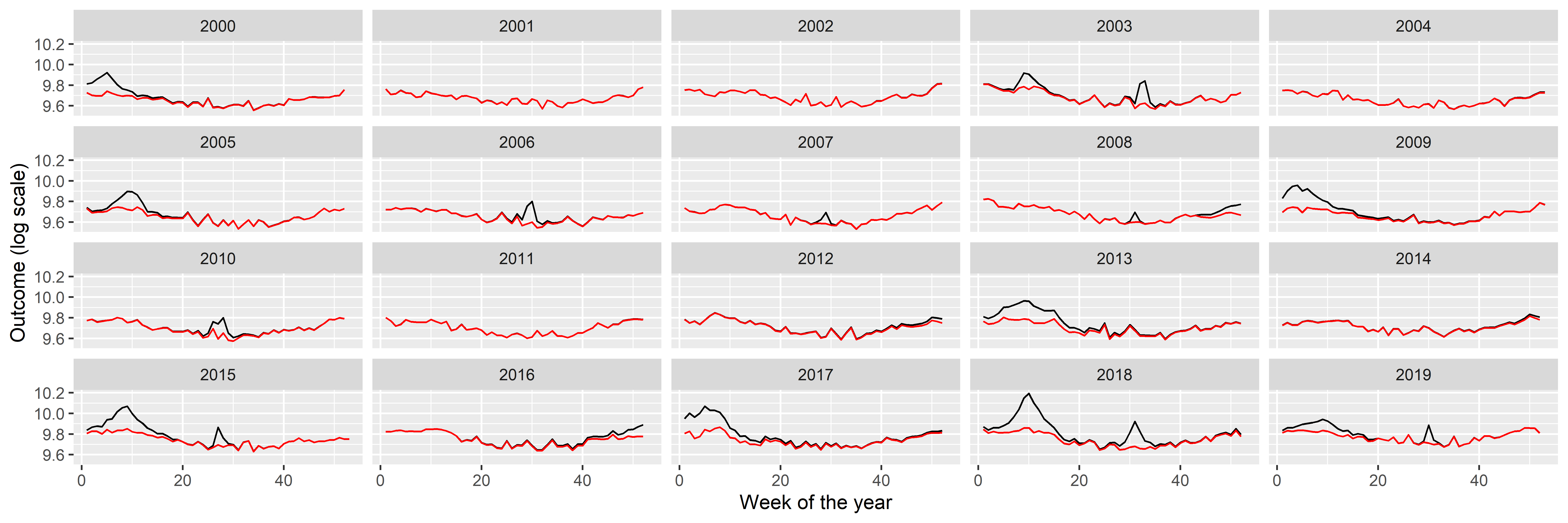


Figure S8: Weekly number of deaths in Germany, separated according to year (black) and with peaks removed (red).

Creating the appropriate model to simulate such peaks is not straightforward: there is stochasticity in the position of the peaks and in its shape, i.e., height and width. (Actually, even the presence of a peak is stochastic.) The following procedure will be used: the presence is generated as a Bernoulli random variate (with the probabilities described above, different for summer and winter), the onset date is uniformly distributed (from 0 to 0.2 in scaled weeks for the winter peak and from 0.5 to 0.7 for the summer peak), i.e., the position itself is random, but the parameters of the underyling distribution are fixed. The $b$ parameter is set to zero (irrespectively of its estimated value, to really capture only the peak, locally – a non-zero $b$ would mean a non-local effect), while $s$ and $a$ are randomly generated for each peak, again, separately for summer and winter peaks. Given the high correlation between $s$ and $a$, not these, but rather $s$ (width) and the amplitude will be generated as a random variate from – independent – uniform distributions. The parameters (minimum and maximum) of the uniform distributions both for $s$ and the amplitude will be considered as a parameter (hyperparameter) of the simulational procedure, just as the $p$ probability of the Bernoulli distribution, all different for summer and winter.

The following table summarizes the parameters:

fit <- mgcv::gam(I(log(outcome)-fitpeak) ~ poly(NumTrend, 2, raw = TRUE) +
 cos(2*pi*WeekScaled) + sin(2*pi*WeekScaled),
 data = RawData2019, method = "REML")

fitSeasonAmplitude <- sqrt(coef(fit)["sin(2 * pi * WeekScaled)"]^2 +
 coef(fit)["cos(2 * pi * WeekScaled)"]^2)
fitSeasonPhase <- atan(-coef(fit)["sin(2 * pi * WeekScaled)"]/coef(fit)[
 "cos(2 * pi * WeekScaled)"])

fittedpars <- setNames(c(coef(fit)[1:3], fitSeasonAmplitude, fitSeasonPhase,
 fitWinterAmplitudeMin, fitWinterAmplitudeMax, fitWinterSigmaMin,
 fitWinterSigmaMax, fitWinterProb,
 fitSummerAmplitudeMin, fitSummerAmplitudeMax, fitSummerSigmaMin,
 fitSummerSigmaMax, fitSummerProb),
 c("TrendConst", "TrendLin", "TrendQuadr",
 "SeasonAmplitude", "SeasonPhase",
 "WinterAmplitudeMin", "WinterAmplitudeMax", "WinterSigmaMin",
 "WinterSigmaMax", "WinterProb",
 "SummerAmplitudeMin", "SummerAmplitudeMax", "SummerSigmaMin",
 "SummerSigmaMax", "SummerProb"))
knitr::kable(data.table(
 Parameter = c("Linear term of the trend", "Constant term of the trend",
 "Quadratic term of the trend", "Amplitude of seasonality (log scale)",
 "Phase of seasonality", "Minimum of winter peak amplitude (log scale)",
 "Maximum of winter peak amplitude (log scale)",
 "Minimum of winter peak width",
 "Maximum of winter peak width", "Probability of winter peak",
 "Minimum of summer peak amplitude (log scale)",
 "Maximum of summer peak amplitude (log scale)",
 "Minimum of summer peak width", "Maximum of summer peak width",
 "Probability of summer peak"),
 Value = fittedpars), digits = 2)

| Parameter | Value |
| --- | --- |
| Constant term of the trend | 10.11 |
| Linear term of the trend | -7.36·10^-5^ |
| Quadratic term of the trend | 3.04·10^-9^ |
| Amplitude of seasonality (log scale) | 0.07 |
| Phase of seasonality | -0.61 |
| Minimum of winter peak amplitude (log scale) | 0.11 |
| Maximum of winter peak amplitude (log scale) | 0.33 |
| Minimum of winter peak width | 8.41 |
| Maximum of winter peak width | 35.46 |
| Probability of winter peak | 0.45 |
| Minimum of summer peak amplitude (log scale) | 0.10 |
| Maximum of summer peak amplitude (log scale) | 0.24 |
| Minimum of summer peak width | 0.86 |
| Maximum of summer peak width | 9.24 |
| Probability of summer peak | 0.40 |
| Size parameter of the negative binomial distribution | 1000 |

saveRDS(fittedpars, "fittedpars.rds")

Given the mechanism described above, the procedure to simulate synthetic datasets can easily be created. Of course, as every calculation is carried out on the log scale, the mean should be exponentiated at the last step.

simdat <- function(TrendConst, TrendLin, TrendQuadr, SeasonAmplitude, SeasonPhase,
 WinterAmplitudeMin, WinterAmplitudeMax, WinterSigmaMin, WinterSigmaMax,
 WinterProb,
 SummerAmplitudeMin, SummerAmplitudeMax, SummerSigmaMin, SummerSigmaMax,
 SummerProb) {

 SimData <- data.frame(date = seq(as.Date("2000-01-03"), as.Date("2023-12-25"), by = 7))
 SimData$NumTrend <- as.numeric(SimData$date)
 SimData$WeekScaled <- lubridate::isoweek(SimData$date)/
 lubridate::isoweek(paste0(lubridate::isoyear(SimData$date), "-12-28"))

 SimData$logmu <- TrendConst + TrendLin*SimData$NumTrend +
 TrendQuadr*SimData$NumTrend^2 + SeasonAmplitude*cos(SimData$WeekScaled*2*pi +
 SeasonPhase)

 SimData$logmu <- SimData$logmu + rowSums(sapply(2000:2023, function(y) {
 if(rbinom(1, 1, WinterProb)==0) return(rep(0, nrow(SimData))) else {
 amplitude <- runif(1, WinterAmplitudeMin, WinterAmplitudeMax)
 sigm <- runif(1, WinterSigmaMin, WinterSigmaMax)
 dcauchy(SimData$NumTrend,
 as.numeric((as.Date(paste0(y, "-01-01")) + runif(1, 0, 0.2)*7*52.25)),
 sigm)*(pi*sigm*amplitude)
 }
 }))

 SimData$logmu <- SimData$logmu + rowSums(sapply(2000:2023, function(y) {
 if(rbinom(1, 1, SummerProb)==0) return(rep(0, nrow(SimData))) else {
 amplitude <- runif(1, SummerAmplitudeMin, SummerAmplitudeMax)
 sigm <- runif(1, SummerSigmaMin, SummerSigmaMax)
 dcauchy(SimData$NumTrend,
 as.numeric((as.Date(paste0(y, "-01-01")) + runif(1, 0.5, 0.7)*7*52.25)),
 sigm)*(pi*sigm*amplitude)
 }
 }))

 SimData$outcome <- rnbinom(nrow(SimData), mu = exp(SimData$logmu), size = 1000)

 SimData
}

Figure S9 illustrates the synthetic data set creation with a single simulation. (Of course, to assess the correctness of the simulation, several realizations have to be inspected.) In addition to the plots already seen, it also gives the autocorrelation function so that it can also be compared. (The simulated outcomes are themselves independent – meaning that effects like the increased probability of a flu season if the previous year did not have one are neglected –, but the trend, seasonality and the peaks induce a correlation structure.)

set.seed(1)

SimData <- as.data.table(do.call(simdat, as.list(fittedpars)))
SimData$Type <- "Simulated"
SimData$Week <- lubridate::isoweek(SimData$date)
SimData$Year <- lubridate::isoyear(SimData$date)

SimDataYearly <- SimData[, .(outcome = sum(outcome)), .(Year, Type)]

p1 <- ggplot(rbind(SimData[Year<=2019], RawData[Year<=2019]),
 aes(x = date, y = outcome, group = Type, color = Type)) + geom_line() +
 labs(x = "Date", y = "Mortality [/week]")

p2 <- ggplot(rbind(SimDataYearly[Year<=2019], RawDataYearly[Year<=2019]),
 aes(x = Year, y = outcome, group = Type, color = Type)) + geom_point() +
 geom_line() + labs(y = "Mortality [/year]")

p3 <- ggplot(rbind(SimData[Year<=2019], RawData[Year<=2019]),
 aes(x = Week, y = outcome, group = Year)) + facet_wrap(~Type) +
 geom_line(alpha = 0.2) + labs(x = "Week of the year", y = "Mortality [/week]")

p4 <- ggplot(rbind(with(acf(RawData$outcome, plot = FALSE),
 data.table(Type = "Actual", acf = acf[, 1, 1],
 lag = lag[, 1, 1])),
 with(acf(SimData$outcome, plot = FALSE),
 data.table(Type = "Simulated", acf = acf[, 1, 1],
 lag = lag[, 1, 1]))),
 aes(x = lag - 1/4 + as.numeric(Type=="Simulated")/2,
 xend = lag - 1/4 + as.numeric(Type=="Simulated")/2, y = acf, yend = 0,
 color = Type)) + geom_line() + geom_point() +
 geom_hline(yintercept = 0, col = "black") +
 labs(x = "Lag", y = "Autocorrelation")

egg::ggarrange(p1, p2, p3, p4, ncol = 1)





Figure S9: From top to bottom: weekly mortalities, yearly mortalities, seasonal pattern and autocorrelation function of the actual German mortality data and a single simulated dataset, 2000-2019.

Several simulations confirm an overall good fit between the actual data set and the simulated ones. Thus, it is now possible to investigate the properties of the mortality prediction on algorithms using simulated datasets, where the actual outcome is known, and the parameters can be varied.

## References

1. Wood SN. Generalized additive models: An introduction with R. Second edition. Boca Raton: CRC Press/Taylor & Francis Group; 2017.

2. Hilbe JM. Negative Binomial Regression. 2nd ed. Cambridge University Press; 2011. Available from: <https://www.cambridge.org/core/product/identifier/9780511973420/type/book>

3. Palshikar G. Simple algorithms for peak detection in time-series. Proc 1st Int Conf Advanced Data Analysis, Business Analytics and Intelligence. 2009.

4. Nelder JA, Mead R. A Simplex Method for Function Minimization. The Computer Journal. 1965;7:308–13. Available from: <https://academic.oup.com/comjnl/article-lookup/doi/10.1093/comjnl/7.4.308>
