# Supplementary material for: Comparing methods to predict baseline mortality for excess mortality calculations
Source: BMC Med Res Methodol. 2023 Oct 18;23:239. doi: 10.1186/s12874-023-02061-w (PMC10585880; doi:10.1186/s12874-023-02061-w)
Supplement: Supplementary file 3 — Additional file 3. Validation through simulation. [file 12874_2023_2061_MOESM3_ESM.docx]

**Additional File 3: Validation through simulation**

Two sets of parameters have to be set up: parameters of the simulation (i.e., parameters of the scenario, on which the methods will be run) and parameters of the methods. They’re set up as given in the main text.

pargridSim <- rbind(as.data.table(t(fittedpars)),
 rbindlist(lapply(1:length(fittedpars), function(i) {
 temp <- as.data.table(t(fittedpars))[rep(1, 5)]
 temp[[names(fittedpars)[i]]] <-
 seq(fittedpars[i]*if(i==1) 0.9 else 0.5,
 fittedpars[i]*if(i==1) 1.1 else 2,
 length.out = 5)
 temp
 })))
pargridSim$SummerProb[pargridSim$SummerProb>1] <- 1
pargridSim$WinterProb[pargridSim$WinterProb>1] <- 1

pargridWHO <- expand.grid(startyear = c(2000, 2005, 2010, 2015), k = c(3, 5, 10, 15))
pargridWHO$parmethod <- paste0("WHO", seq_len(nrow(pargridWHO)))
pargridAI <- expand.grid(startyear = c(2000, 2005, 2010, 2015), invtkpy = c(4, 5, 7, 12))
pargridAI <- merge(pargridAI, data.table(invtkpy = c(4, 5, 7, 12),
 tkpy = c("1/4", "1/5", "1/7", "1/12")))
pargridAI$parmethod <- paste0("AI", seq_len(nrow(pargridAI)))
pargridAI$tkpy <- factor(pargridAI$tkpy, levels = c("1/12", "1/7", "1/5", "1/4"))
pargridAverage <- data.frame(startyear = c(2000, 2005, 2010, 2015, 2019))
pargridAverage$parmethod <- paste0("Average", seq_len(nrow(pargridAverage)))
pargridLin <- data.frame(startyear = c(2000, 2005, 2010, 2015))
pargridLin$parmethod <- paste0("Lin", seq_len(nrow(pargridLin)))

One thousand simulation will be run for each parameter of the scenario, and for each of the 1000 simulated data set all 4 methods with all possible parameters of the methods will be evaluated. To increase the speed, simulations will be run in parallel. (The problem is embarrassingly parallel, as different simulations are completely independent of each other [1].)

if(!file.exists("predLongs.rds")) {
 cl <- parallel::makeCluster(parallel::detectCores()-1)
 parallel::clusterExport(cl, c("simdat", "pargridSim", "pargridWHO", "pargridAI",
 "pargridAverage", "pargridLin"))

 for(r in 1:10) {
 pred <- do.call(rbind, parallel::parLapply(cl, 1:100, function(j) {
 do.call(rbind, lapply(1:nrow(pargridSim), function(k) {
 SimData <- do.call(simdat, as.list(pargridSim[k, ]))
 SimData$Year <- lubridate::isoyear(SimData$date)

 predWHO <- sapply(1:nrow(pargridWHO), function(i)
 predict(mgcv::gam(outcome ~ s(NumTrend, k = pargridWHO$k[i]) +
 s(WeekScaled, bs = "cc"),
 data = SimData[SimData$Year>=pargridWHO$startyear[i]&
 SimData$Year<=2019,],
 family = mgcv::nb(), method = "REML"),
 newdata = SimData[SimData$Year>=2020,], type = "response"))

 predAI <- sapply(1:nrow(pargridAI), function(i)
 with(excessmort::compute_expected(
 cbind(SimData[SimData$Year>=pargridAI$startyear[i],], population = 1),
 exclude = seq(as.Date("2020-01-01"), max(SimData$date), by = "day"),
 frequency = nrow(SimData)/(as.numeric(diff(range(SimData$date)))/365.25),
 trend.knots.per.year = 1/pargridAI$invtkpy[i], verbose = FALSE),
 expected[date>=as.Date("2019-12-30")]))

 predAverage <- sapply(1:nrow(pargridAverage), function(i)
 predict(mgcv::gam(outcome ~ s(WeekScaled, bs = "cc"),
 data = SimData[SimData$Year>=pargridAverage$startyear[i]&
 SimData$Year<=2019,],
 family = mgcv::nb(), method = "REML"),
 newdata = SimData[SimData$Year>=2020,], type = "response"))

 predLin <- sapply(1:nrow(pargridLin), function(i)
 predict(mgcv::gam(outcome ~ NumTrend + s(WeekScaled, bs = "cc"),
 data = SimData[SimData$Year>=pargridLin$startyear[i]&
 SimData$Year<=2019,],
 family = mgcv::nb(), method = "REML"),
 newdata = SimData[SimData$Year>=2020,], type = "response"))

 setNames(data.frame(j, k, SimData[SimData$Year>=2020, c("date", "outcome")],
 predWHO, predAI, predAverage, predLin, row.names = NULL),
 c("rep", "parsim", "date", "outcome", pargridWHO$parmethod,
 pargridAI$parmethod, pargridAverage$parmethod, pargridLin$parmethod))
 }))
 }))

 pred$rep <- (r-1)*100 + pred$rep
 saveRDS(pred, paste0("pred_", r, ".rds"))
 }

 parallel::stopCluster(cl)

 pred <- rbindlist(lapply(1:10, function(r) readRDS(paste0("pred_", r, ".rds"))))
 saveRDS(pred, "pred.rds")

 pred$Year <- lubridate::isoyear(pred$date)
 pred$date <- NULL

 predYearly <- pred[, lapply(.SD, sum), .(rep, parsim, Year)]

 predLongs <- lapply(
 list(WHO = pargridWHO, AI = pargridAI, Average = pargridAverage,
 Lin = pargridLin),
 function(pg)
 merge(merge(melt(predYearly[, c("rep", "parsim", "outcome", "Year", pg$parmethod),
 with = FALSE],
 id.vars = c("rep", "parsim", "outcome", "Year"),
 variable.name = "parmethod"), pg),
 data.table(parsim = 1:76,
 parsimName = factor(c("Base", rep(names(fittedpars), each = 5)),
 levels = c("Base", names(fittedpars))),
 parsimValue = c("Base", rep(paste0("#", 1:5),
 length(fittedpars)))), by = "parsim"))

 saveRDS(predLongs, "predLongs.rds")
} else predLongs <- readRDS("predLongs.rds")

## References

1. Matloff NS. The art of R programming: Tour of statistical software design. San Francisco: No Starch Press; 2011.
