# Supplementary material for: Comparing methods to predict baseline mortality for excess mortality calculations
Source: BMC Med Res Methodol. 2023 Oct 18;23:239. doi: 10.1186/s12874-023-02061-w (PMC10585880; doi:10.1186/s12874-023-02061-w)
Supplement: Supplementary file 4 — Additional file 4. Directly comparing the errors in simulation runs. [file 12874_2023_2061_MOESM4_ESM.docx]

**Additional File 4: Directly comparing the errors in simulation runs**

As different methods were evaluated on the same simulated dataset for each simulation, it is possible to compare not only the averages, but directly compare the errors themselves. Figure S10 shows direct comparison between the best parametrization of the WHO’s method and the Acosta-Irizarry method for 200 randomly selected simulations in each scenario, with 2015 as the starting year. In the base case scenario, Acosta-Irizarry performed better in 59.8% of the cases.


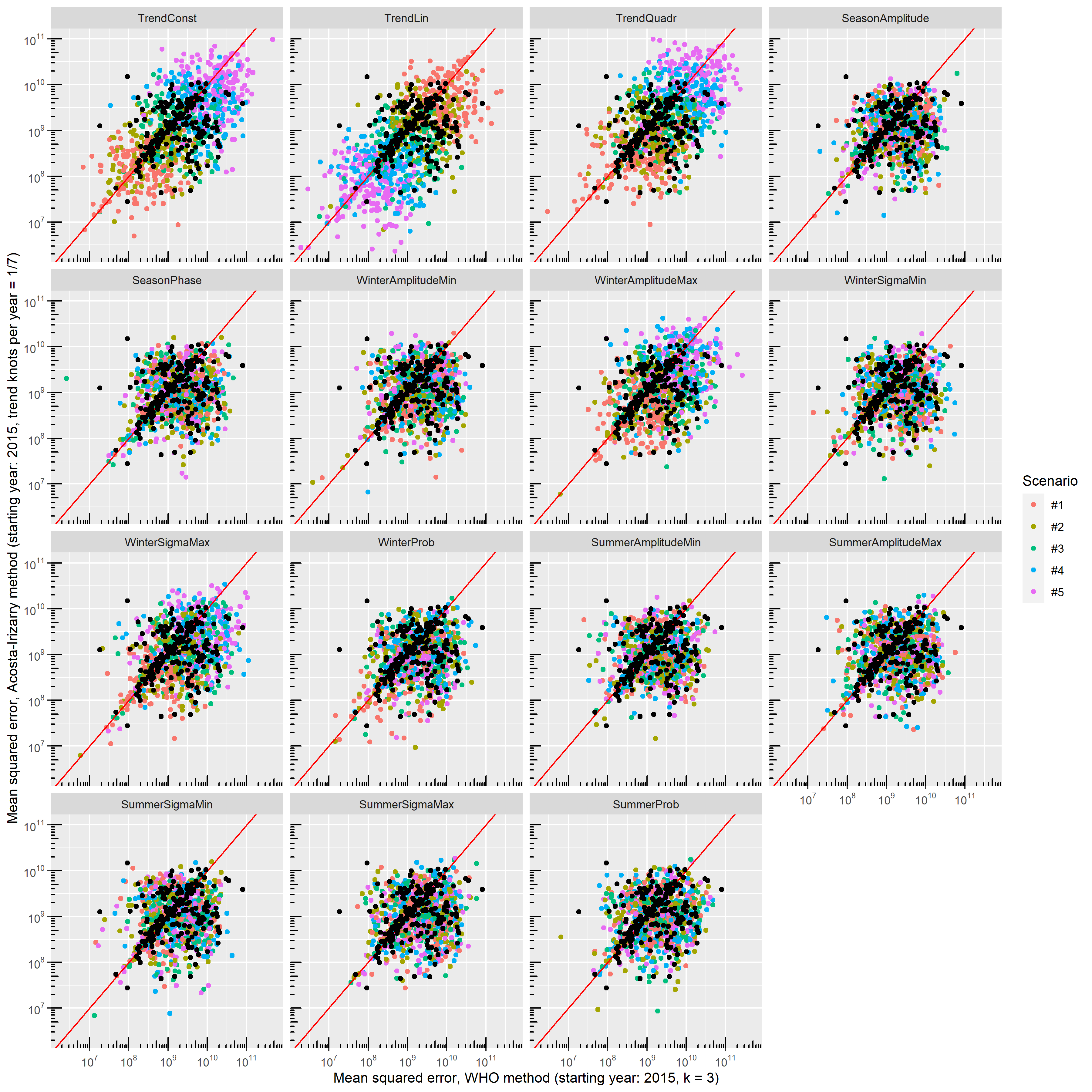


Figure S10: Errors – squared distance of the predicted outcome from its true value – of the WHO’s method and the Acosta-Irizarry method (under best parametrization) on the same simulated datasets for 200 randomly selected simulations with different scenarios; black dots indicate the base case scenario, scenario #1 to #5 represent varying the parameter shown on the panel from half of its base case value to twice (with the exception of the constant term where it is varied from 90% to 110%), and probabilities are limited to be below 100%.
